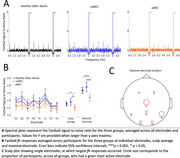# A passive and objective measure of recognition memory in Mild Cognitive Impairment using Fastball memory assessment

**DOI:** 10.1002/alz70863_110467

**Published:** 2025-12-23

**Authors:** George Stothart, Sophie Alderman, Oliver P Hermann, Elizabeth Coulthard

**Affiliations:** ^1^ University of Bath, Bath UK; ^2^ University of Bath (The), Bath, Avon UK; ^3^ Bristol Medical School, University of Bristol, Bristol UK

## Abstract

**Background:**

As viable pharmacotherapies and blood biomarkers emerge for dementia treatment and screening there remains a great need for accurate, sensitive biomarkers of cognitive function. We have previously demonstrated that Fastball, a new EEG method for the passive and objective measurement of recognition memory that requires no behavioural memory response or comprehension of the task, is sensitive to cognitive dysfunction in Alzheimer's disease. Here we present new evidence that Fastball is sensitive to amnestic dysfunction in an earlier stage of the dementia lifecourse, Mild Cognitive Impairment.

**Method:**

53 MCI patients and 54 healthy older adult controls completed a 3‐minute Fastball task in which they passively viewed rapidly presented images while EEG captured their automatic ability to differentiate between images based on previous exposure. They also completed neuropsychological assessments of memory (DMS‐48), sustained attention (Psychomotor Vigilance Task) and general cognitive function (ACE‐iii). Participants were re‐tested after one year to establish the test‐retest reliability of Fastball in healthy older adults, and the sensitivity of Fastball to cognitive decline in MCI patients, over a one year period.

**Results:**

Amnestic MCI patients showed significantly reduced Fastball responses compared to non‐amnestic MCI patients (*p* = 0.001, Cohen's d = 0.98). and healthy older adult controls (*p* = 0.005, Cohen's d = 0.64). Regression analyses showed that Fastball EEG responses were selectively predictive of neuropsychological measures of recognition memory and not attentional function. At year one follow‐up Fastball showed moderate to good test‐retest reliability in healthy older adult controls, and the six MCI‐dementia converters showed a trend for lower Fastball responses at baseline which will be confirmed with further longitudinal assessment.

**Conclusion:**

Fastball is further validated as a viable method for testing recognition memory in cognitively impaired populations. We have demonstrated that it is selectively predictive of memory dysfunction and not other cognitive functions such as attention. It is passive, non‐invasive, quick to administer and uses cheap, scalable EEG technology. Fastball is a viable functional biomarker that can help to advance cognitive assessment in MCI.